# Exploring Underrepresentation: The Role of Diversity Statements in Ophthalmology Residency Programs

**DOI:** 10.7759/cureus.56569

**Published:** 2024-03-20

**Authors:** Mahad Rehman, Amber Nanni, Sruthi Suresh, Ibrahim Saleh, Sujata Dalal, Masuma Firoz, Monica Patel, Brandon Georges, Ahmed S Rehman, Karanjit S Kooner

**Affiliations:** 1 Ophthalmology, University of Texas Southwestern Medical Center, Dallas, USA; 2 Ophthalmology, Veterans Affairs North Texas Health Care System, Dallas, USA

**Keywords:** medical education, diversity statements, underrepresented minorities, ophthalmology residency programs, diversity

## Abstract

Introduction: The underrepresentation of underrepresented minorities (URMs) in the medical field, particularly in ophthalmology, poses a critical challenge to achieving diversity and equity. While URMs constitute 19% of medical school attendees, their presence is markedly lower in ophthalmology residency programs and among practicing ophthalmologists. This study seeks to investigate the prevalence of diversity statements on ophthalmology residency program websites and their role in the underrepresentation of URMs within the field.

Methods: This observational, cross-sectional study analyzed the websites of 126 ophthalmology residency programs listed on the San Francisco (SF) Match website. Diversity statements were categorized based on their inclusion of specific underrepresented groups (race or ethnicity, gender, sexual orientation, and disability) and analyzed for correlation with program characteristics. Descriptive statistics and Chi-squared tests were utilized to assess the prevalence of diversity statements and their association with program size, ranking, geographical location, and institutional nature.

Results: Of the 126 programs analyzed, 21 (16.7%) had diversity statements specific to the ophthalmology residency program, and 115 (91.3%) featured institutional-level diversity statements. Race or ethnicity was the most commonly addressed category in diversity statements (75.3%), followed by gender (65.9%), sexual orientation (61.1%), and disability (53.2%). Statistical analyses revealed no significant correlation between program size and the presence of diversity statements. However, higher-ranked programs were more likely to mention sexual orientation and disability. Significant differences were observed at the institutional level, with public institutions more likely to include specific diversity categories.

Conclusion: The study highlights a significant disparity in the presence and focus of diversity statements across ophthalmology residency programs. Despite a high prevalence of institutional-level diversity statements, program-specific initiatives are lacking, particularly in addressing disability inclusion. The findings suggest a need for a more comprehensive and targeted effort to address underrepresentation in ophthalmology.

## Introduction

Racial and ethnic representation in the medical field varies considerably across specialties [1,2]. Studies have shown that primary care specialties such as family medicine, internal medicine, and obstetrics and gynecology are the most racially and ethnically diverse specialties, whereas surgical fields such as ophthalmology, plastic surgery, and orthopedic surgery are among the least diverse [1,2]. While underrepresented minorities (URMs) make up 19% of medical school attendees, they make up only 6.3% of ophthalmology residency programs and 7.2% of current practicing ophthalmologists [3]. Despite this significant disparity, the underlying factors contributing to this underrepresentation in ophthalmology remain largely unexplored.

To address this gap, this study seeks to better understand the underlying reasons for the disparity in URM representation within the field of ophthalmology. We aim to investigate the prevalence of diversity statements on residency program websites and categorize these statements based on the inclusion of specific underrepresented groups such as race, ethnicity, gender, sexual orientation, and disability. Moreover, we will contextualize these statements within various program characteristics, such as geographical region, size, ranking, and institutional nature (public or private), offering a novel perspective not extensively explored in previous literature. We hypothesize that the absence of diversity statements on ophthalmology residency program websites plays a significant role in this disparity.

## Materials and methods

An observational, cross-sectional study was conducted on the websites of ophthalmology residency programs from the San Francisco (SF) Match website. As all the data were gathered from publicly accessible online materials, institutional review board approval was deemed unnecessary for this study. All data were gathered between April and May 2023.

Inclusion criteria

Our study considered the 126 ophthalmology programs that were listed on the SF Match website maintained by the American Academy of Ophthalmology as of 2023. Programs with missing or malfunctioning hyperlinks and those requiring a login to view their diversity statements or policies were excluded.

Data collection

For those programs that met our data collection, team members categorized diversity statements into four groups based on the content and location of the statements: (1) statements specifically referencing the ophthalmology residency program, linked or listed on the residency program website, (2) statements within the ophthalmology department, linked or listed on the department website, (3) statements within the larger institution where the program is affiliated, linked or listed on the program or department websites, and (4) statements within the larger institution that were not directly linked or listed and were manually identified through a separate search.

Importantly, statements of nondiscrimination were not considered as substitutes for diversity statements. Additionally, among programs with diversity statements, the team also assessed whether statements mention specific diversity categories, such as race or ethnicity, gender, sexual orientation, and disability. References to racial or ethnic groups included both direct mentions and labels highlighting their lower representation in the medical field. Furthermore, diversity statements that highlighted the inclusion of women in ophthalmology were considered indicative of gender diversity inclusion. We further evaluated whether program size, ranking, geographic location, or institutional nature (public or private) was associated with differences in the presence of diversity statements. All findings were recorded on a Microsoft Excel document (Microsoft Corporation, Redwood, WA). To guarantee the privacy and safety of the information, methods were implemented including password protection for the Excel file and restricting access to authorized personnel only.

Statistical analysis

Descriptive statistics were used to summarize the prevalence of diversity statements within the ophthalmology residency programs. We quantified the number and percentage of programs that included diversity statements specific to the ophthalmology residency program, department, and institution, as well as those that mentioned specific diversity categories such as race or ethnicity, gender, sexual orientation, and disability.

Statistical analyses were also performed to explore correlations between these diversity-related factors and various program characteristics: program size, ranking, geographic location, and the public or private nature of the institution. For assessing correlations with program size and ranking, point-biserial correlation coefficients were calculated, providing a measure of the strength and direction of association between these continuous variables and the categorical diversity-related factors. The significance of these correlations was determined based on their p-values, with a threshold of p <0.05 denoting statistical significance.

To examine the relationship between geographic location or the type of institution (public or private) and diversity-related factors, Chi-squared tests were utilized. These tests evaluated whether the frequency distribution of diversity statements and specific diversity categories differed based on geographic location or institution type. The Chi-squared values and corresponding two-tailed p-values were reported, again using a significance threshold of p <0.05.

## Results

Our study assessed 126 ophthalmology residency programs for the presence and content of diversity statements. As seen in Table 1, only 21 (16.7%) programs contained diversity statements that were specific to the ophthalmology residency program. A slightly higher percentage, 31 programs (24.6%), had diversity statements at the departmental level. The most common location for diversity statements was at the institutional level, with 115 programs (91.3%) featuring such statements. When considering the specific categories of diversity mentioned in these statements, race or ethnicity was the most commonly addressed, featuring in 95 programs (75.3%). Gender was mentioned in 83 programs (65.9%), sexual orientation in 77 programs (61.1%), and disability in 67 programs (53.2%).

**Table 1:** Prevalence of diversity statements across ophthalmology programs

Components of Diversity Statements	Number of Programs (N=126)	Percentage of Programs from Total (%)
Ophthalmology residency program	21	16.7%
Department diversity statement	31	24.6%
Institution diversity statement	115	91.3%
Race or ethnicity	95	75.3%
Gender	83	65.9%
Sexual orientation	77	61.1%
Disability	67	53.2%

We also explored correlations based on program size, ranking, geographic location, and whether an institution is public or private. For program size, no significant correlation was found with the presence of diversity statements or specific diversity categories (Table 2). However, rankings were significantly correlated with mentions of sexual orientation and disability in diversity statements, suggesting that higher-ranked programs (which have smaller ranking numbers) more frequently mention these categories (Table 3). There were no significant findings regarding rankings and the presence of other diversity statements or categories, such as race or ethnicity and gender.

**Table 2:** Correlations with ophthalmology program size

Comparisons	Correlation	p-Value
Ophthalmology residency program diversity statement vs size	0.0342	0.7049
Department diversity statement vs size	0.0220	0.8077
Institution diversity statement vs size	-0.0154	0.8643
Race or ethnicity vs size	0.0173	0.8483
Gender vs size	0.0514	0.5688
Sexual orientation vs size	0.0315	0.7276
Disability vs size	0.0773	0.3916

**Table 3:** Correlations with ophthalmology program ranking *Statistically significant p-value.

Comparisons	Correlation	p-Value
Ophthalmology residency program diversity statement vs ranking	-0.0782	0.3859
Department diversity statement vs ranking	-0.1300	0.1484
Institution diversity statement vs ranking	-0.1223	0.1744
Race or ethnicity vs ranking	-0.1575	0.0795
Gender vs ranking	-0.1588	0.0769
Sexual orientation vs ranking	-0.2217	0.0130*
Disability vs ranking	-0.2568	0.0038*

Geographic location did not show a significant correlation with the presence of diversity statements or the inclusion of diversity categories within those statements (Table 4). Lastly, the analysis did not find a significant correlation between the type of institution (public or private) and the presence of ophthalmology residency program-specific or departmental diversity statements. However, there were significant differences at the institutional level and in mentions of specific diversity categories such as race or ethnicity, gender, sexual orientation, and disability between public and private institutions (Table 5).

**Table 4:** Differences based on ophthalmology program location

Comparisons	Chi-Squared Value	p-Value
Ophthalmology residency program diversity statement vs location	1.8189	0.6108
Department diversity statement vs location	3.5336	0.3164
Institution diversity statement vs location	3.4567	0.3264
Race or ethnicity vs location	2.0839	0.5552
Gender vs location	1.8299	0.6084
Sexual orientation vs location	0.9390	0.8160
Disability vs location	0.5402	0.9100

**Table 5:** Differences across public vs private ophthalmology programs *Statistically significant p-value.

Comparisons	Chi-Squared Value	p-Value
Ophthalmology residency program diversity statement vs public or private	9.16	0.3290
Department diversity statement vs public or private	6.05	0.6421
Institution diversity statement vs public or private	22.72	0.0037*
Race or ethnicity vs public or private	17.08	0.0292*
Gender vs public or private	16.43	0.0367*
Sexual orientation vs public or private	17.09	0.0292*
Disability vs public or private	17.21	0.0280*

## Discussion

Prevalence and location of diversity statements

Our study provides a comprehensive analysis of the prevalence and content of diversity statements in 126 ophthalmology residency programs. Notably, a minority of programs (16.7%) had diversity statements specific to the ophthalmology residency itself. The number marginally increased to 24.6% when considering diversity statements at the departmental level. These findings suggest that the majority of ophthalmology residency programs do not place a specific focus on diversity at the program or departmental level.

On the contrary, a large majority of programs (91.3%) included diversity statements at the institutional level. While this could be interpreted as a positive sign of broad institutional commitment to diversity, it also raises questions about the translation of such commitments to individual departments and programs. The absence of program-specific diversity statements indicates a lack of tailored approaches to fostering diversity within ophthalmology residencies that may play a significant role in the lack of representation among URMs in the field of ophthalmology.

Categories of diversity addressed

While ophthalmology residency programs demonstrate some attention to diversity, notable gaps exist across categories. Race or ethnicity, although most frequently cited in diversity statements (75.3%), is still absent in a significant portion of programs, calling attention to the underrepresentation of minorities in the medical field. Gender and sexual orientation, mentioned in 65.9% and 61.1% of statements, respectively, also reveal opportunities for more targeted inclusivity efforts, particularly as they remain significant issues in healthcare. Most glaringly, disability is the least-mentioned category, included in only 53.2% of diversity statements. This is particularly concerning given that over 25% of US adults identify as having a disability, but only 3% of physicians do, underlining the need for greater focus on disability inclusivity. In summary, each diversity category presents unique challenges for inclusion in ophthalmology, and the absence of specific categories in a considerable number of programs signals a need for more comprehensive and targeted commitments to diversity.

Size and location

No significant correlations were found between the size of the programs and the presence of diversity statements or specific diversity categories. This suggests that the commitment to diversity, as evidenced through diversity statements, does not vary substantially with program size.

Similarly, our analysis of geographic location revealed no significant correlations with the presence of diversity statements or inclusion of diversity categories. This finding implies that the emphasis on diversity within ophthalmology residency programs does not appear to be influenced by geographic region, suggesting a uniformly distributed approach to diversity across different areas of the country.

Ranking

Contrastingly, the analysis of program rankings presented significant findings. Notably, higher-ranked programs showed a significant correlation with the increased mention of sexual orientation and disability in their diversity statements. This could imply that programs with higher rankings are more attentive or committed to these specific aspects of diversity. It is possible that these programs, often being more visible and subject to greater scrutiny, may feel a heightened responsibility to address and include a broader range of diversity categories.

The lack of significant correlations in other areas, such as the presence of diversity statements related to race or ethnicity and gender with program ranking, raises questions about the uniformity and depth of diversity initiatives across different programs. It suggests that while some aspects of diversity are being recognized and addressed, others may not be receiving equal attention.

Public vs private institutions

This study also revealed differences based on the public or private nature of the institution. Our results showed no statistical significance in the presence of program-specific or department-level diversity statements between public and private institutions. However, statistically significant differences were noted for institutional-level diversity statements and mentions of specific diversity categories, such as race, gender, sexual orientation, and disability.

Given the statistically significant findings, one may infer that public institutions have a more formalized or mandated approach to diversity, especially in the identified categories. However, the absence of significance at the program or departmental level across public and private institutions may indicate an overall lack of focus on diversity in ophthalmology residencies, irrespective of the type of institution.

Comparison to other specialties

In comparison to other medical specialties, the prevalence of residency-specific diversity and inclusion statements in ophthalmology is notably lower, at just 16.7% as shown in Table 6. This is notably less than other specialties like General Surgery (39.8%), Dermatology (34.6%), Internal Medicine (30%), and Orthopedic Surgery (29.1%). Interestingly, ophthalmology residency programs have fewer diversity statements, even when compared to specialties like Orthopedic Surgery, which has comparable representation levels for URMs.

**Table 6:** Percent of programs with residency-specific diversity and inclusion statement across specialties From the papers [4-12].

Specialty	% of Programs with Residency-Specific Diversity and Inclusion Statement
General Surgery	39.80%
Dermatology	34.60%
Internal Medicine	30%
Orthopedic Surgery	29.10%
Ophthalmology (2022 study)	24%
Radiology	18%
General, Thoracic, and Vascular Surgery	18.60%
Physical Medicine and Rehabilitation	17%
Ophthalmology (this study)	16.7%

Our study also found a lower percentage of ophthalmology programs had diversity statements compared to a previous similar study performed in 2022. Possible explanations for this discrepancy may include differences in inclusion criteria between the two studies or year-over-year variations in program priorities within the ophthalmology specialty.

Why diversity is important in ophthalmology

In the US, URM groups face significant disparities in eye health. Black and Hispanic youth, for instance, experience blindness and visual impairment rates that are 1.9 and 1.5 times higher than their White counterparts, respectively [12]. Similarly, Black individuals encounter glaucoma rates nearly double that of White individuals, yet there remains a substantial gap in glaucoma testing for the Black community [12,13]. Recent research further indicates that Black individuals experience a 1.4 times higher rate of diabetic retinopathy than White individuals [12].

Enhanced diversity in healthcare is not just a matter of representation; it directly influences the quality of care. According to LaVeist and Pierre, a diverse healthcare setting achieves five crucial outcomes: it boosts patient satisfaction and trust, enhances cultural competency in patient-provider interactions, broadens access to health services for minority patients, increases healthcare provisions in underserved areas, and offers a more comprehensive racial and ethnic perspective in research [14]. Consistently, diverse healthcare teams have been associated with superior clinical results and improved patient care quality [14].

The imperative for diversity has gained further urgency in light of the 2023 Supreme Court rulings. Decisions that invalidated race-conscious admissions programs [15], allowed businesses to refuse services for same-sex weddings [16], and reversed a proposal to abolish $430 billion in student loan debt [17] could deter URMs from pursuing medical careers. Similarly, recent bills passed by lawmakers in Texas and Florida ban diversity, equity, and inclusion (DEI) offices, programs, and training at publicly funded universities, presenting a further challenge for the implementation of diversity-enhancing strategies in these states [18,19]. These developments underscore the urgency for ophthalmology residency programs to prioritize diversity, both as a moral obligation and a strategy for optimal patient care outcomes.

Strategies for improving diversity in ophthalmology residency programs

DEI strategies are critical components of a robust and equitable healthcare system. Ophthalmology residency programs play a pivotal role in shaping the future of eye care, and to ensure that this future is representative and culturally sensitive, it is imperative to implement effective strategies for improving diversity within these programs.

There is a strong link between faculty diversity and state demographics which highlights the challenges faced by medical schools in low-minority states, suggesting the need for increased efforts to attract and retain minority faculty members [20]. One of the fundamental strategies for enhancing diversity in ophthalmology residency programs is proactive recruitment efforts. Previous studies have indicated that extramural rotations positively correlated with a student's ability to match into a specialty since it provided the applicant an opportunity to show interest in a program, demonstrate abilities, and receive a letter of recommendation from a faculty member [21]. Programs can collaborate with underrepresented minority ophthalmology associations, participate in pipeline programs, and engage in outreach efforts at institutions with diverse student populations [20]. The American Academy of Ophthalmology website offers various Minority Ophthalmology Research Mentorship programs (Rabb-Venable Research Program, Howard Hughes Medical Institute (HHMI) Medical Research Fellows Program, National Eye Interest (NEI) Internships and Student Programs, Research to Prevent Blindness (RPB)/Allergan Foundation Medical Student Eye Research Fellowship) and pairing students or young physicians with LGBTQ+ mentors. Cultivating relationships with medical students from underrepresented backgrounds is equally vital; these relationships not only encourage interest in ophthalmology but also provide a supportive network. Implicit bias training is another essential strategy. By educating faculty, interviewers, and selection committees about unconscious biases, programs can ensure that the selection process is fair and unbiased, allowing for a more diverse pool of candidates [22].

Financial barriers often deter talented individuals from pursuing residency programs in low-minority areas. In the 2016-2017 academic year, it was discovered that a greater percentage of underrepresented medical students, particularly African Americans, graduated with educational debt. To be precise, 81.5% of White students carried education debt compared to a higher 93.4% of their Black counterparts [23]. Additionally, Black Medical students are found to accumulate more debt compared to Whites, 17.1% and 10.3%, respectively, in 2016-2017 accumulated more than $300,000 of debt [23]. To address this, ophthalmology programs may consider offering diversity scholarships and financial support tailored to underrepresented minority residents. By monitoring and evaluating these strategies, ophthalmology residency programs may not only increase diversity and inclusion but also contribute to a more inclusive and culturally competent healthcare workforce that can better serve the diverse needs of patients [24].

Limitations

Our study primarily focuses on information available on departmental websites, overlooking other avenues through which DEI efforts could be communicated, such as social media or direct correspondence with applicants. This focus could skew our understanding of the true state of DEI initiatives within these programs. Additionally, the predetermined criteria used to assess the presence and quality of diversity statements have not been validated for the purposes of this study. This lack of validation could potentially introduce bias into our findings and interpretations. Lastly, as mentioned earlier, states such as Texas and Florida have implemented recent bills banning DEI offices, programs, and training at publicly funded universities. These initiatives may make it difficult to assess the efforts made by such schools to foster diversity among ophthalmology residents. However, the data collection for this study was conducted prior to the implementation of these laws in both states, minimizing their impact on our findings.

Directions for future studies

Given the observed limitations, future studies could expand the scope beyond departmental websites. Surveys or interviews with current residents could offer a more nuanced understanding of DEI in practice rather than theory. Tracking longitudinal changes in diversity statements and comparing them to actual demographic changes in these programs could also be instructive. As mentioned earlier we hope to perform similar studies to assess the change in the prevalence of diversity and inclusion information on ophthalmology residency program websites on a yearly basis. Lastly, given the possibility of shifts in DEI (diversity, equity, and inclusion) efforts due to recent legislative changes in Texas and Florida, this study could be pivotal as an initial assessment of the current state of these initiatives. Moving forward, it may become a valuable reference for evaluating how these laws influence the presence of diversity statements on program websites.

## Conclusions

In conclusion, our analysis of 126 ophthalmology residency programs reveals significant disparities in the presence and focus of diversity statements at various institutional levels. This study aimed to delve deeper into the underlying reasons for the disparity in URM representation within ophthalmology. Our findings strongly support the hypothesis that the absence or insufficiency of diversity statements on ophthalmology residency program websites may be a significant contributor to this disparity. While many programs have diversity statements at the institutional level, the lack of program-specific initiatives and the underrepresentation of those with disabilities in these narratives particularly stand out. This reinforces the importance of ophthalmology programs to improve their commitment to diversity by embedding these principles within their structures and practices. Only with dedicated, sustained effort can we hope to see a more diverse, representative, and inclusive future for the field of ophthalmology.
